# Focal dose escalation using FDG-PET-guided intensity-modulated radiation therapy boost for postoperative local recurrent rectal cancer: a planning study with comparison of DVH and NTCP

**DOI:** 10.1186/1471-2407-10-127

**Published:** 2010-04-07

**Authors:** Keiichi Jingu, Hisanori Ariga, Tomohiro Kaneta, Yoshihiro Takai, Ken Takeda, Lindel Katja, Kakutaro Narazaki, Takahiro Metoki, Keisuke Fujimoto, Rei Umezawa, Yoshihiro Ogawa, Kenji Nemoto, Masashi Koto, Masatoshi Mitsuya, Naruhiro Matsufuji, Shoki Takahashi, Shogo Yamada

**Affiliations:** 1 Department of Radiation Oncology, Tohoku University School of Medicine, Sendai, Japan; 2 Department of Diagnostic Radiology, Tohoku University School of Medicine, Sendai, Japan; 3 Department of Radiation Oncology, Yamagata University School of Medicine, Yamagata, Japan; 4 Department of Radiooncology, Heidelberg University, Heidelberg, Germany; 5 Department of Accelerator Physics and Engineering, National Institute of Radiological Sciences, Chiba, Japan

## Abstract

**Background:**

To evaluate the safety of focal dose escalation to regions with standardized uptake value (SUV) >2.0 using intensity-modulated radiation therapy (IMRT) by comparison of radiotherapy plans using dose-volume histograms (DVHs) and normal tissue complication probability (NTCP) for postoperative local recurrent rectal cancer

**Methods:**

First, we performed conventional radiotherapy with 40 Gy/20 fr. (CRT 40 Gy) for 12 patients with postoperative local recurrent rectal cancer, and then we performed FDG-PET/CT radiotherapy planning for those patients. We defined the regions with SUV > 2.0 as biological target volume (BTV) and made three boost plans for each patient: 1) CRT boost plan, 2) IMRT without dose-painting boost plan, and 3) IMRT with dose-painting boost plan. The total boost dose was 20 Gy. In IMRT with dose-painting boost plan, we increased the dose for BTV+5 mm by 30% of the prescribed dose. We added CRT boost plan to CRT 40 Gy (*summed plan 1*), IMRT without dose-painting boost plan to CRT 40 Gy (*summed plan 2*) and IMRT with dose-painting boost plan to CRT 40 Gy (*summed plan 3*), and we compared those plans using DVHs and NTCP.

**Results:**

D_mean _of PTV-PET and that of PTV-CT were 26.5 Gy and 21.3 Gy, respectively. V_50 _of small bowel PRV in *summed plan 1 *was significantly higher than those in other plans ((*summed plan 1 *vs. *summed plan 2 *vs. *summed plan 3*: 47.11 ± 45.33 cm^3 ^vs. 40.63 ± 39.13 cm^3 ^vs. 41.25 ± 39.96 cm^3^(p < 0.01, respectively)). There were no significant differences in V_30_, V_40_, V_60_, D_mean _or NTCP of small bowel PRV.

**Conclusions:**

FDG-PET-guided IMRT can facilitate focal dose-escalation to regions with SUV above 2.0 for postoperative local recurrent rectal cancer.

## Background

Although positron emission tomography using ^18^F-fluorodeoxyglucose (FDG-PET) has become widely used for diagnosis of various malignant tumors, the spatial resolution of PET images alone is not high and it is difficult to determine anatomical sites in detail. However, this problem has been solved by the use of a combined PET/CT system, which enables both PET and CT images to be obtained at almost the same time and at the same position.

Local recurrence rates of rectal cancer after surgery including dissection of lateral nodes have been reported to be about 9~12% in Japan [1-3], and the prognosis after local recurrence is poor. In the case of local recurrence, the best salvage treatment for achieving long-term local control and survival is total pelvic exenteration with distal sacrectomy. The 5-year overall survival rate in patients after R0 resection has been reported to be 30~40% [4,5]. Since about half of the patients with local recurrent rectal cancer die due to only local lesions without distant metastasis [6], local control would be beneficial for survival. However, extended surgery is not widely used because of high morbidity and mortality rates. Moreover, it has been pointed out that total pelvic exenteration reduces the quality of life of patients. Furthermore, Tepper et al. reported that only 34% of patients with locally or distantly recurrent rectal cancer could receive a potentially curative resection [7]. In Japan, due to the lower rate of local recurrence after surgery alone, induction radiotherapy is not performed in most patients [8]. And, based on SEER, over 30% of patients with advanced-stage rectal cancer in the United States also did not undergo radiation therapy [9]. Therefore, external body radiotherapy is one of the most widely used therapies and provides good palliation of pain in 50~80% of patients with postoperative local recurrence; however, it has a poor survival benefit [10]. We have been performing conventional irradiation for postoperative local recurrent lesions with a total dose of 60 Gy (2 Gy/fraction · 5 fractions/week), but we have considered that dose escalation is necessary to cure patients because rectal cancer has many hypoxic fractions [11]. In fact, some studies have revealed that local failure rate after radiotherapy alone decreased with increasing irradiation dose [12,13]. However, dose escalation with conventional radiotherapy is difficult due to the location of critical organs (e.g, small bowel) around the lesion.

Huebner et al. showed by a meta-analysis that the sensitivity, specificity and accuracy of FDG-PET for local recurrent rectal cancer were 94.5%, 97.7% and 95.9%, respectively [14]. FDG-PET is superior to conventional modalities (e.g, CT and MRI) for distinguishing between local recurrence and postoperative scar. There have been several reports recently on the usefulness of FDG-PET for radiotherapy planning in lung cancer and head and neck cancer. FDG-PET has been reported to be useful for delineation of target gross tumor volume (GTV) or clinical target volume (CTV).

We performed FDG-PET/CT planning in 12 patients with postoperative local recurrent rectal cancer during conventional radiotherapy at 40 Gy.

As a preclinical study, we planned focal dose escalation to high FDG uptake regions in those 12 patients with intensity-modulated radiation therapy (IMRT) in the radiotherapy planning system, and we compared the IMRT plans with conventional radiotherapy plans in dose-volume histograms (DVHs) and normal tissue complication probability (NTCP).

The purpose of the present study is to evaluate the safety of focal dose escalation to regions with standardized uptake value (SUV) above 2.0 using IMRT in DVH and NTCP in patients with postoperative locoregional recurrent rectal cancer

## Methods

### Criteria for eligibility

Eligibility criteria were as follows: (1) postoperative locoregional recurrent rectal cancer, (2) unresectable, (3) age between 20 and 79 years, (4) Karnofsky Performance Status (KPS) score of > = 60, (4) without distant metastasis, (5) tumor is grossly measurable, and (6) no serious medical or psychologic conditions precluding safe administration of treatment.

### Radiotherapy

A linear accelerator (Clinac 23EX (VARIAN Medical Systems, Palo Alto, CA), 6 or 15 MV) was used as the X-ray source.

First, we performed radiotherapy planning using CT with contrast medium for 12 patients with postoperative locoregional recurrent rectal cancer. All target volumes were outlined slice by slice on the treatment-planning CT images. GTV was defined as the gross extent of the tumor shown by imaging as well as physical examination, CTV was defined as GTV plus a 10-mm circular margin for potential microscopic spread, and planning target volume (PTV) was defined as CTV plus a 5-mm circular margin to account for organ motion and patient setup errors. Additionally, we attached a 5-mm leaf margin to PTV. The patients were prescribed 40 Gy in 20 fractions with the dose prescribed to the isocenter (CRT 40 Gy) using a median of 4 (range 3-4) coplanar irradiation fields.

Next, we performed FDG-PET/CT with a carbon graphite flat tabletop in the supine position for radiotherapy planning at 40 Gy in the same 12 patients. The images obtained by CT and PET were sent to the radiation therapy planning system as DICOM data, and the CT and PET images were fused using DICOM information. Residual gross extent of the tumor shown in CT images at 40 Gy was defined as GTV2, CTV-CT was defined as GTV2 plus a 5-mm circular margin, and PTV-CT was defined as CTV-CT plus a 5-mm circular margin. We defined the regions with standardized uptake value (SUV) above 2.0 as biological target volume (BTV) and BTV+5-mm circular margin as PTV-PET. Our radiotherapy planning system could show the degree of FDG accumulation with not SUV but Bq/ml in PET images. If the total dose of FDG administered to the patient and the patient's body weight are known, we can define regions with an arbitrary range of SUVs even in our radiotherapy planning system by adjusting the window level and range. In the present study, we delineated BTV under the condition showing SUV of 2.0 to 20.0.

**Figure 1:**
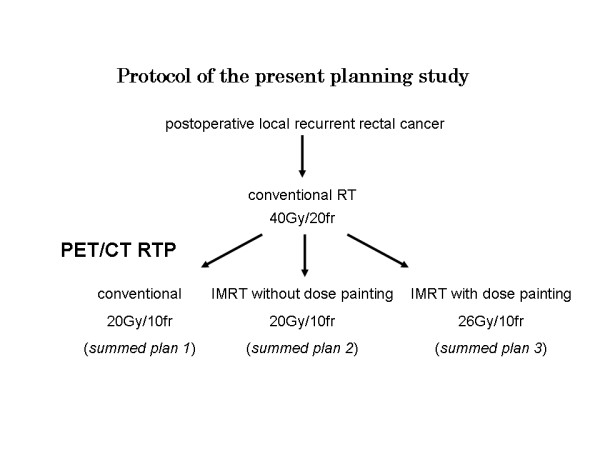
**Pattern diagram of the present planning study**.

We made three boost plans for each patient: 1) conventional radiotherapy plan (CRT boost plan), 2) IMRT plan not using dose painting (IMRT without dose-painting boost plan), and 3) IMRT plan using dose painting (IMRT with dose-painting boost plan) (Figure 1). The fractional dose of radiotherapy was 2.0 Gy with normalization at 95% of PTV-CT, and the total boost dose was 20.0 Gy. In IMRT dose-painting boost plan, we increased the dose of PTV-PET by 30% of the prescribed dose (2.6 Gy/fraction, total 26.0 Gy) using dose-painting.

We defined the small bowel as the organ at risk (OAR) because the small bowel is the most vulnerable to radiation in pelvic organs, and we carefully delineated the whole small bowel in the abdomen and pelvis, preferably with the colon, bladder or other organs with reference to CT using contrast medium. The planning organ at risk volume (PRV) was margined with 5 mm to the OAR as with the PTV margin.

IMRT plans were generated using Varian Eclipse (Helios IMRT) Workstations. The beam arrangement consisted of seven coplanar non-colinear fields (30°, 80°, 130°, 180°, 230°, 280°, 330°), and delivery of IMRT was carried out using the sliding window technique with a 15 MV linear accelerator equipped with a dynamic multileaf collimator. Our primary inverse planning objectives were as follows: (1) uniform dose of 20 Gy to PTV-CT (relative weight, w = 15), (2) maximal irradiated dose (Dmax) of small bowel PRV <20 Gy (w = 20) (total Dmax of small bowel PRV <60 Gy), (3) dose received by 5% (D5) of small bowel PRV <15 Gy (w = 8) (total D5 of small bowel PRV <55 Gy), and in the IMRT with dose-painting boost plan, we added (4) dose received by 95% (D95) of PTV-PET >26 Gy (w = 10) (total D95 of PTV-PET >66 Gy, which means normalized 2-Gy-equivalent biologically effective dose, 67.3 Gy, calculated using an alpha/beta value of 10).

IMRT planning for this study was theoretical and was not used for treatment. All of the patients underwent conformal radiation therapy to a total dose of 60~66 Gy (2.0 Gy/fraction/day).

**Figure 2:**
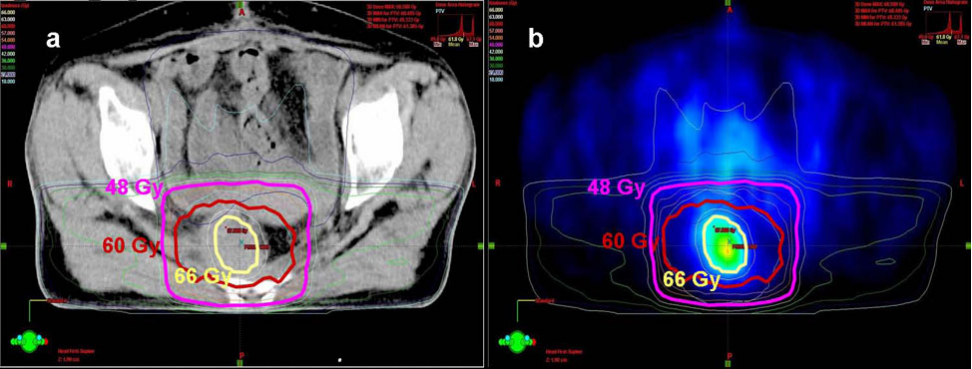
**Dose distribution of *summed plan 3 *(summed plan of CRT 40Gy and IMRT with dose-painting boost plan) in a patient**. a): dose distribution in CT, b): dose distribution in FDG-PET.

We added each boost plan to CRT 40 Gy (*summed plan 1*: CRT 40 Gy + CRT boost plan, *summed plan 2*: CRT 40 Gy + IMRT without dose-painting boost plan, *summed plan 3*: CRT 40 Gy + IMRT with dose-painting boost plan) (Figure 2) and we compared those plans using DVHs and NTCP of small bowel PRV.

The differences in dose distribution between IMRT without dose-painting boost plan and IMRT with dose-painting boost plan were evaluated by the conformity index (C.I.), which we defined as the ratio of the volume irradiated with 95% of the prescribed dose to PTV-CT.

### NTCP

NTCP was calculated using the Lyman-Kutcher-Burman model.(1)(2)

Irradiation of fractional volume *v *= V/V_ref _corresponds to parameter TD50 (*v*) for 50% complication probability, given by the following power law:(3)

V_ref _is the total volume of the organ.

With inhomogeneous irradiation, there are multiple partial volume irradiations to different doses. The effects of partial volume irradiations are computed according to the Kutcher-Burman (K-B) effective-volume dose-volume histogram reduction scheme [15]. The K-B method satisfies the consistency requirement that partitioning a uniform irradiation of the entire volume into multiple partial volume irradiations to the same dose results in the same NTCP. The organ dose is described as independent fractional volume elements *v*_j_, (*j *= 1,... *k*),  = 1, irradiated to doses d_j_. Then, with the K-B algorithm, an effective fractional volume V_eff(j) _defined as follows is computed for each *v*_j_. The effective fractional volume is the volume that, when irradiated to reference dose d_ref_, would give the same complication probability in the Lyman model as the actual fractional volume *v*_j _irradiated to dose d_j_. If *v*_j _were the only fractional volume irradiated, then from equality of effect and Eq. (B), it would follow that(4)

In the K-B method, Eq. (D) is applied to all fractional subvolumes. In the present study, d_ref _was chosen to be the maximum dose D_max_. The total effective fractional volume receiving the reference dose is calculated as follows:(5)

using *v *= V_eff _in Eq. (C) and D = d_ref _= D_max _in Eq. (B)

The parameters used to calculate small bowel obstruction and perforation were *n *= 0.15, *m *= 0.16, and TD_50 _= 55 [16,17].

### Chemotherapy

For all of the 12 patients, S-1 60 mg/m^2 ^was given orally twice daily (within 30 minutes after morning and evening meals) for 2 weeks, followed by a drug-free interval of one week (one cycle) concomitant with radiation therapy. Chemotherapy was not performed for a period of at least 4 weeks before initiation of radiation therapy in any of the patients.

### FDG-PET

PET scans were performed 1 hour after administration of ^18^F-fluorodeoxyglucose at a dose of 3.1 MBq/kg using a Biograph PET/CT scanner (Siemens, Hoffman Estates, IL) under the condition of more than 4 hours of fasting. A transmission scan was performed for attenuation correction before the emission scans (using a computed tomography scan). Seven bed positions were used for emission scans, with an acquisition time of 2 minutes per position. For radiotherapy planning, PET/CT scans were performed in the same posture as that for treatment on a flat carbon-fiber table top. The PET images were reconstructed with an ordered-subset expectation maximization (OSEM) iterative reconstruction algorithm.

For semiquantitative analysis of increased FDG uptake lesions, SUV based on body weight (g) was calculated and converted into a value based on lean body mass:

The blood glucose levels of all patients before scans were less than 150 mg/dl.

### Statistical analysis

Statistical significance was defined as a value of p < 0.05 in the present study. SPSS software for Windows version 11.0 (SPSS Inc, Chicago, IL) was used for all calculations. Multiple pairwise comparisons were performed by using one-way analysis of variance *t*-test with the Bonferroni method.

### Ethics

The present study protocol was reviewed and approved by the Ethics Committee of Tohoku University Graduate School of Medicine (approval number, 2007-418), and informed consent was obtained from all patients before radiation therapy.

## Results

**Figure 3:**
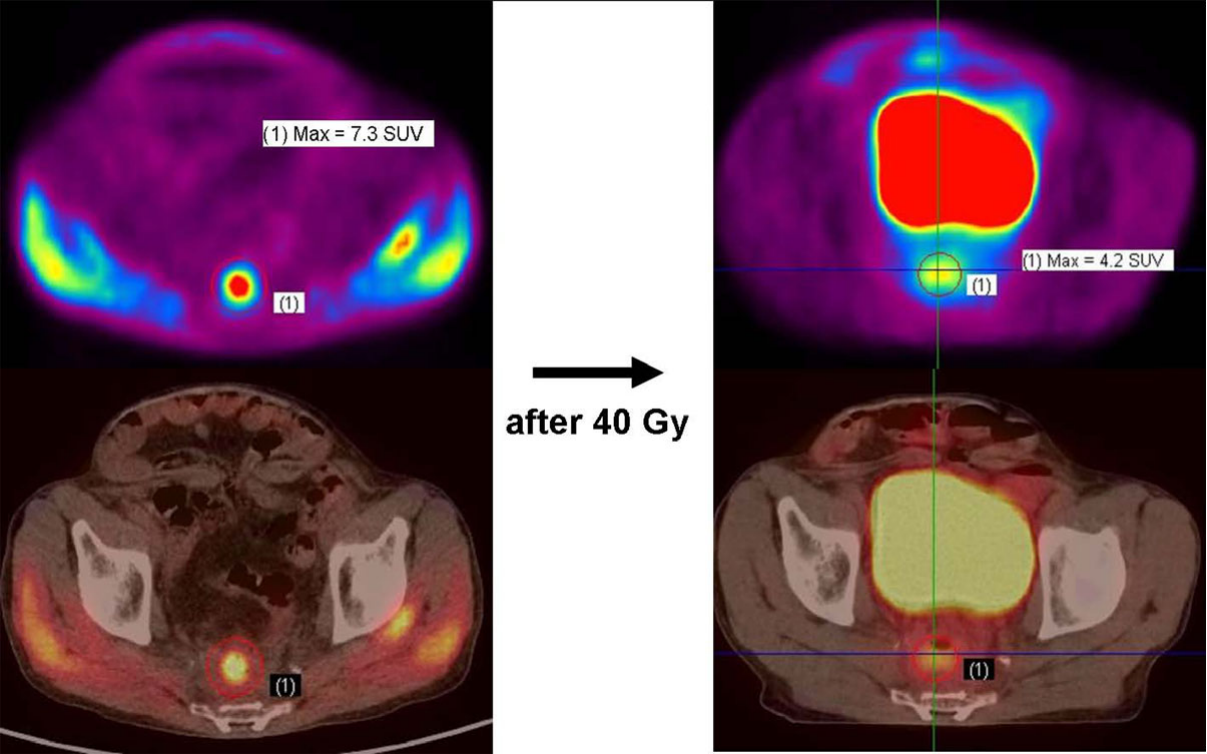
**Change in FDG accumulation**. This patient had anastomatic recurrence with 7.3 SUVmax before radiation therapy. After 40 Gy, the accumulation was decreased by 3.1 SUVmax.

The results of comparison of the plans in all 12 patients are shown in Additional file 1; Table S1. Even with the fusion method using DICOM information, there were no significant displacements between PET images and CT images. We did not need to fuse them manually again. The locations of the highest level of FDG accumulation after 40 Gy in local recurrent regions were almost the same as those before radiation therapy in the 12 patients, but maximal SUV decreased significantly from 6.84 ± 3.25 before radiation therapy to 5.14 ± 2.81 at 40 Gy (p = 0.035, Wilcoxon's test). Figure 3 shows change in FDG accumulation caused by irradiation of 40 Gy in a patient with anastomotic recurrence. In the present study, although there was no significant difference between GTV and GTV2 (GTV vs. GTV2: 87.52 ± 63.06 cm^3 ^vs. 79.66 ± 57.80 cm^3^, p = 0.141), there was a significant difference between GTV2 and BTV (GTV2 vs. BTV: 79.66 ± 57.80 cm^3 ^vs. 11.12 ± 21.92 cm^3^, p < 0.001) (Additional file 1; Table S2). In the IMRT with dose-painting boost plan, mean irradiated dose (D_mean_) of PTV-PET and that of PTV-CT were 26.5 ± 0.8 Gy and 21.3 ± 0.8 Gy, respectively.

With regard to the volume of small bowel PRV receiving 50 Gy or more (V_50_), there were significant differences between *summed plan 1 *and *summed plan 2 *and between *summed plan 1 *and *summed plan 3 *(*summed plan 1 *vs. *summed plan 2 *vs. *summed plan 3*: 47.11 ± 45.33 cm^3 ^vs. 40.63 ± 39.13 cm^3 ^vs. 41.25 ± 39.96 cm^3 ^(p < 0.01, respectively)) (Additional file 1; Table S2).

**Figure 4:**
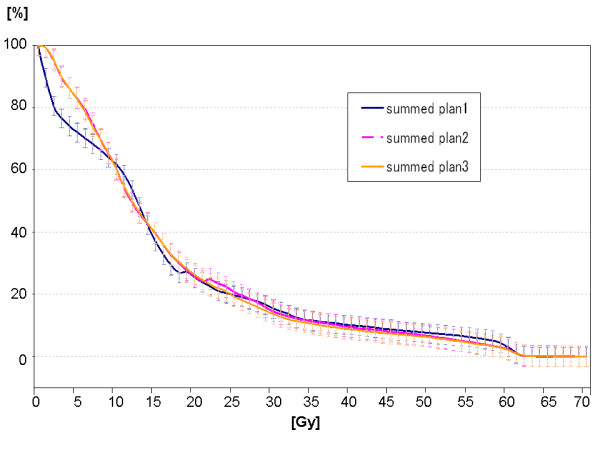
**Mean DVH (whole curve) of small bowel PRV in each summed plan**.

**Figure 5:**
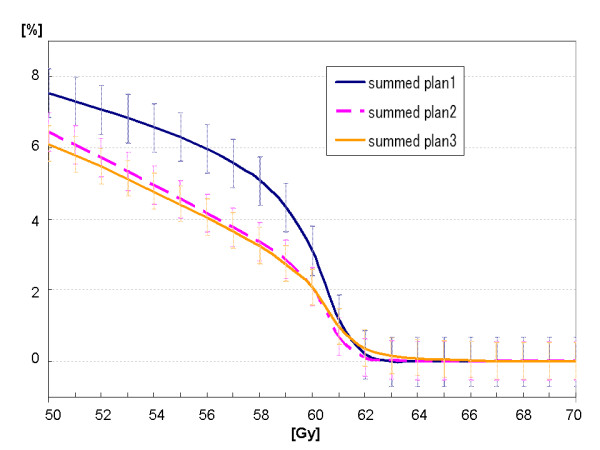
**Mean DVH (focused on high dose area (50 to 70 Gy)) of small bowel PRV in each summed plan**.

With regard to the volume of small bowel PRV receiving 60 Gy or more (V_60_), 40 Gy or more (V_40_), 30 Gy or more (V_30_) and D_mean _of small bowel PRV, there were no significant differences (*summed plan 1 *vs. *summed plan 2 *vs. *summed plan 3*: V_60_, 19.76 ± 23.67 cm^3 ^vs. 13.65 ± 18.88 cm^3 ^vs. 14.52 ± 19.18 cm^3^; V_40_, 77.32 ± 64.21 cm^3 ^vs. 71.33 ± 60.20 cm^3 ^vs. 72.55 ± 61.59 cm^3^; V_30_, 121.18 ± 119.6 cm^3 ^vs. 113.62 ± 99.69 cm^3 ^vs. 116.88 ± 104.94 cm^3^; D_mean_, 16.1 ± 5.8 Gy vs. 16.4 ± 5.6 Gy vs. 16.6 ± 5.8 Gy (n.s.)) (Figure 4 and 5, Additional file 1; Table S2).

**Figure 6:**
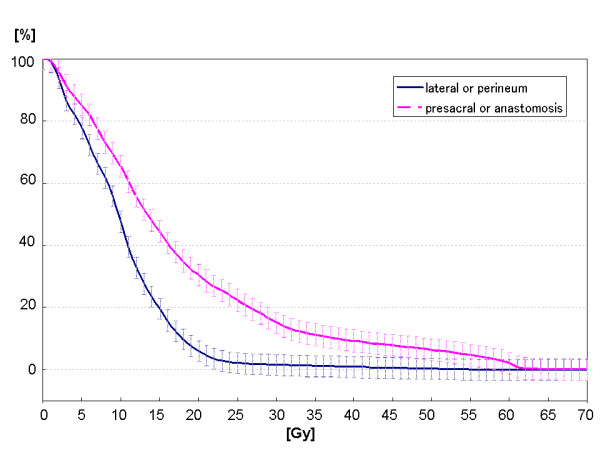
**Mean DVH of small bowel PRV of *summed plan 3 *in patients with lateral pelvic lymph node metastasis or perineum recurrence and that in patients with presacral or anastomostic recurrence**. In patients with lateral pelvic lymph node metastasis or perineum recurrence, the mean DVH of small bowel PRV of *summed plan 3 *was significantly lower than that in patients with presacral or anastomostic recurrence.

Focal dose escalation using dose-painting slightly but significantly increased maximal irradiated dose (D_max_) of small bowel PRV(*summed plan 1 *vs. *summed plan 2 *vs. *summed plan 3*: 55.0 ± 16.0 Gy vs. 54.9 ± 15.3 Gy vs. 57.4 ± 16.3 Gy (p < 0.01, respectively)); however NTCP of small bowel PRV was not significantly increased even using focal dose escalation (*summed plan 1 *vs. *summed plan 2 *vs. *summed plan 3*: 5.10 ± 5.66% vs. 3.78 ± 4.19% vs. 4.09 ± 4.62%) (Additional file 1; Table S2). In the 4 patients with lateral pelvic lymph node metastasis or perineum recurrence, there were no significant differences in D_max _or NTCP of small bowel PRV (*summed plan 1 *vs. *summed plan 2 *vs. *summed plan 3*: D_max_, 41.5 ± 23.9 Gy vs. 42.5 ± 23.4 Gy vs. 43.8 ± 24.2 Gy; NTCP, 4.45 ± 8.84% vs. 2.95 ± 5.87% vs. 3.30 ± 6.57%). In 8 patients with presacral or anastomostic recurrence, D_max _of small bowel PRV of *summed plan 3 *was significantly higher than that of *summed plan 2 *(p = 0.006) but was not significantly higher than that of *summed plan 1 *(n.s.) (*summed plan 1 *vs. *summed plan 2 *vs. *summed plan 3*: 61.8 ± 0.6 Gy vs. 61.1 ± 1.1 Gy vs. 64.2 ± 3.0 Gy); however, IMRT could significantly decrease NTCP of small bowel PRV. There was no significant difference in NTCP of small bowel PRV between *summed plan 2 *and *summed plan 3 *(*summed plan 1 *vs. *summed plan 2 *vs. *summed plan 3: *5.42 ± 4.05% vs. 4.19 ± 3.56% vs. 4.49 ± 3.82%, p < 0.005, respectively). The mean DVH of small bowel PRV of *summed plan 3 *in patients with lateral pelvic lymph node metastasis or perineum recurrence and that in patients with presacral or anastomostic recurrence are shown in Figure 6.

In the present study, although D_max _of small bowel PRV of *summed plan 3 *was slightly higher than that of *summed plan 1 *or *summed plan 2*, V_50 _of small bowel PRV could be reduced by IMRT, and V_30_, V_40_, V_60_, D_mean _and NTCP were not increased even using focal dose escalation.

There was also no significant difference in C.I. between IMRT without dose-painting and IMRT with dose-painting (IMRT without dose-painting boost plan vs. IMRT with dose-painting boost plan: 1.33 ± 0.10 vs. 1.29 ± 0.61 (p = 0.115)).

## Discussion

To our knowledge, there are few reports on PET-guided IMRT for lower gastrointestinal cancer. The reasons why we used this planning method for patients with local recurrent rectal cancer were 1) FDG-PET enabled a recurrent tumor to be distinguished from postoperative scar, 2) FDG-PET could reveal the region with higher malignancy activity and 3) it was not necessary to consider large inter- and intra-fractional motions because of adhesion due to the operation.

There have been several reports on PET/CT radiotherapy planning in lung cancer and in head and neck carcinoma. This planning method has been reported to be useful for radiotherapy to delineate target volume. With regard to FDG, there is evidence that FDG-avid regions of a tumor show increased radioresistance in vitro [18,19] and hypoxia in vivo [20]. Therefore, FDG-PET/CT is also useful for radiotherapy planning to detect the region with high residual potency in GTV to be given priority for treatment with a high dose.

It is difficult to clearly show threshold accumulation between malignancy and non-malignancy by FDG-PET because there is usually inflammation around a malignant tumor, there is penumbra of high accumulation and the normal gut tube has slightly high uptake of FDG. There have been many reports on contouring target volume according to 40~50% of maximal SUV value, source-background ratio, and arbitrary SUV value in some malignant tumors [21-25]; however, it remains inconclusive. Bayne et al. pointed out that SUV value had problems with accuracy and reproducibility [26]. In the present study, although we used an arbitrary SUV of 2.0, we consider that it is not a clear border between malignancy and non-malignancy but a region with relatively high malignant potency and with resistance to radiation at 40 Gy including a subclinical margin like CTV margin. Although SUV of 2.5~3.0 was used as a threshold value between malignancy and non-malignancy in many previous studies, we used SUV of 2.0 as the threshold value for BTV based on the fact that patients in the present study had already been irradiated with 40 Gy and based on the fact that Haberkorn et al. reported the mean SUV of recurrent rectal cancer after radiotherapy with 40 Gy to be 1.8 [27].

Furthermore, in the present study, since normal tissues around the GTV were also irradiated with 40 Gy, the possibility that radiation-induced inflammation masked a residual malignant tumor must also be considered. For other tumors such as head and neck cancer and lymphoma [28,29], chemoradiation-induced inflammatory response causes sufficient numbers of false-positive results limiting PET being performed less than 2 months after chemoradiation. It may be inappropriate to use FDG-PET for radiation planning during radiation therapy. Recently, chemotherapy consisting of 5-FU or Capecitabine with or without the addition of Oxaliplatinum has commonly been performed for recurrent rectal cancer. Also, in the present study, all patients underwent concomitant and/or previous chemotherapy with radiation therapy. Findlay et al. mentioned the so-called flare phenomenon that occurs at 1~2 weeks after the initiation of chemotherapy and that can be observed as a marked increase in FDG metabolism in lesions that show response later [30]. We may also have to investigate the appropriate thresholds of FDG accumulation for BTV for each type of chemotherapy. However, in rectal cancer, many investigators revealed that the positive predictive value of FDG-PET assessment of therapy response during or soon after chemoradiation was very high and was not significantly limited by post-chemoradiation changes [31]. The timing of FDG-PET after chemoradiation for the most accurate assessment of tumor response in rectal cancer is controversial. Further larger prospective surveys of the time courses of tumor FDG uptake during and after chemoradiation in rectal cancer are required.

There are other major problems regarding the use of PET/CT for radiation therapy planning: misalignment of the fusion of PET and CT images due to body movement, bowel peristalsis and difference in volume of urine between the transmission scan and emission scan as well as artifacts due to FDG in urine, so-called "hot urine". These problems can be resolved to a large extent by overnight fasting before PET/CT and by starting the emission scan from the position of the pelvis. Moreover, in the present study, a 5-mm circular margin was attached to each target volume and OAR; however, it might not be sufficient to cover such misalignment. It is necessary to investigate such misalignment using on-line imaging (e.g., cone-beam CT) before clinical application.

In the present study, since V_30_, V_40_, V_60_, D_mean _and NTCP of small bowel PRV were not increased and V_50 _of small bowel PRV could be reduced due to the differences between GTV2 and BTV, focal dose escalation by 6 Gy to regions with SUV above 2.0 using IMRT with dose-painting boost for postoperative local recurrent rectal cancer is considered to be safe. FDG-PET-guided IMRT has the possibility of improving local control of postoperative local recurrent rectal cancer without increasing the risk of radiation injury of small bowel PRV. However, although NTCP which reflects account all the DVH data was not increased, D_max _of small bowel PRV in the summed plan using focal dose escalation was significantly higher than that in other summed plans. While the differences in mean D_max _of small bowel PRV between *summed plan 3 *and the other plans were only about 2.5 Gy in the present study, D_max _of small bowel PRV in *summed plan 3 *was more than 65.0 Gy in 4 of the 8 patients with anastomotic or presacral recurrence, and NTCP in *summed plan 3 *in 2 of the 4 patients was more than 10%. Since it is known that the small bowel is a "serial organ" and that the dose at which probability of obstruction or perforation is 50% within 5 years after treatment (TD50/5) of the small bowel is 55 Gy [32], although NTCP shows that focal dose escalation is acceptable, dose escalation by only 6 Gy from 60 Gy even using PET-guided IMRT is relatively risky. Therefore, if the region of high FDG accumulation is near the OARs, it might be necessary to reduce the degree of dose escalation and/or reduce the volume to increase irradiation dose (e.g., lesion with SUV > 2.5). Alternatively, using IMRT from the beginning of radiotherapy, using a belly board, and inserting a spacer between the recurrent tumor and OARs may further facilitate dose escalation without increasing the risk of radiation injury. When PTV-PET overlaps PRV, we may have to further modify the irradiation dose setting of the overlapping part.

Rectal cancer is known to have many hypoxic fractions [11]. Some studies have provided evidence that hypoxia has a negative impact on tumor response to radiation and other methods of therapy [33-36]. Although we used FDG for radiotherapy planning in this study to determine the region with high tumor cell density, it may be more important for improving the effect of radiotherapy for rectal cancer to determine the hypoxic regions. There are some tracers for detecting a hypoxic region (e.g., [^18^F]Fluoromisonidazole-3-fluoro-1-(2'-nitro-1'-imidazolyl)-2-propanol ([^18^F]FMISO), Cu-diacetyl-bis(N4-methylthiosemicarbazone (Cu-ATSM) and 1-(2-fluoro-1-[hydroxymethyl]ethoxy)methyl-2-nitroimidazole ([^18^F]FRP170)) [37-39]. Although Lin et al. have already reported the effectiveness in head and neck cancer [40], increasing the irradiation dose with IMRT to the hypoxic region may also be effective for treating postoperative recurrent rectal cancer.

## Conclusions

Our findings suggest that FDG-PET/CT-guided IMRT can facilitate focal dose escalation to regions with SUV above 2.0 while providing normal tissue protection in patients with postoperative local recurrent rectal cancer. However, we do not recommend routine clinical use of focal dose escalation using FDG-PET/CT-guided IMRT. In cases in which the region of high FDG accumulation is near the OARs, careful radiotherapy planning is necessary. Based on the results of this planning study, we will start a clinical phase I/II study of focal dose escalation using PET-guided IMRT for patients with postoperative local recurrent rectal cancer in our institution.

## Competing interests

The authors declare that they have no competing interests.

## Authors' contributions

KJ, ST, YO, KN, HA and SY participated in the design of the study. KJ and YT performed the statistical analysis. KJ, TK, TM and LK conceived of the study and participated in its design and coordination. RU and YO helped to draft the manuscript. HA, KT, KN, KF and MK acquired data. MM and NM verified and calculated DVH and NTCP. All authors read and approved the final manuscript.

## Pre-publication history

The pre-publication history for this paper can be accessed here:

http://www.biomedcentral.com/1471-2407/10/127/prepub

## Supplementary Material

Additional file 1 Supplementary tables. Click here for file
