# Supplementary material for: Focal dose escalation using FDG-PET-guided intensity-modulated radiation therapy boost for postoperative local recurrent rectal cancer: a planning study with comparison of DVH and NTCP
Source: BMC Cancer. 2010 Apr 7;10:127. doi: 10.1186/1471-2407-10-127 (PMC2858110; doi:10.1186/1471-2407-10-127)
Supplement: Additional file 1 — Supplementary tables. [file 1471-2407-10-127-S1.DOC]

**Table 1: Results of comparison of the plans in all 12 patients**

| **patient** | **GTV§ (cm^3^)** | **GTV2* (cm^3^)** | **BTV† (cm^3^)** | **V60Gy　（cm^3^)** | | | **V50Gy　(cm^3^)** | | | **V40Gy　(cm^3^)** | | | **V30Gy　(cm^3^)** | | | **Dmean　(Gy)** | | | **Dmax　(Gy)** | | | **NTCP　(%)** | | |
| --- | --- | --- | --- | --- | --- | --- | --- | --- | --- | --- | --- | --- | --- | --- | --- | --- | --- | --- | --- | --- | --- | --- | --- | --- |
|  |  |  |  | **plan 1** | **plan 2** | **plan 3** | **plan 1** | **plan 　2** | **plan 3** | **plan 1** | **plan 2** | **plan 3** | **plan 1** | **plan 2** | **plan 3** | **plan 1** | **plan 2** | **plan 3** | **plan 1** | **plan 2** | **plan 3** | **plan 1** | **plan 2** | **plan 3** |
| **1**‡ | 70.8 | 66.9 | 4.0 | 0 | 0 | 0 | 0 | 0 | 0 | 0 | 0 | 0 | 0 | 0 | 0 | 7.0 | 7.9 | 7.9 | 18.3 | 20.9 | 21.4 | 0.00 | 0.00 | 0.00 |
| **2**‡ | 152.5 | 162.1 | 6.8 | 0.17 | 0.02 | 0.01 | 1.12 | 0.95 | 0.68 | 6.6 | 9.5 | 8.6 | 11.7 | 16.6 | 15.8 | 6.8 | 9.1 | 8.2 | 61.0 | 61.3 | 60.8 | 0.07 | 0.06 | 0.05 |
| **3** | 30 | 28.7 | 0.7 | 17.5 | 10.6 | 10.6 | 57.9 | 55.2 | 54.7 | 90.1 | 85.6 | 84.9 | 153.6 | 181.5 | 179.7 | 17.3 | 19.6 | 19.6 | 61.4 | 61 | 62.7 | 3.75 | 3.28 | 3.23 |
| **4** | 43.5 | 41.9 | 4.6 | 14.7 | 12.9 | 15.9 | 59.5 | 59.5 | 53.6 | 167.0 | 168.4 | 171.0 | 280.0 | 290.9 | 305.5 | 23.3 | 25 | 25.4 | 61.6 | 62.5 | 65.3 | 9.36 | 8.03 | 8.72 |
| **5** | 95.9 | 43.8 | 7.1 | 65.2 | 62.4 | 61.9 | 142.2 | 124.4 | 127.0 | 172.7 | 162.0 | 164.5 | 386.2 | 262.7 | 280.4 | 23.8 | 23 | 23.3 | 61.4 | 61.9 | 67.8 | 11.96 | 10.18 | 10.94 |
| **6** | 90.2 | 85.7 | 3.0 | 0.52 | 0 | 0 | 7.3 | 6.0 | 6.0 | 16.4 | 16.6 | 16.5 | 24.8 | 29.0 | 28.9 | 12.5 | 13.7 | 13.6 | 61.0 | 58.7 | 59.3 | 0.32 | 0.17 | 0.15 |
| **7**‡ | 72.6 | 71.8 | 3.3 | 64.2 | 37.0 | 40.9 | 106.0 | 84.5 | 88.6 | 115.0 | 98.8 | 100.2 | 133.2 | 126.8 | 128.2 | 23.8 | 23 | 23.4 | 63.1 | 64.1 | 68.3 | 17.71 | 11.75 | 13.16 |
| **8** | 25 | 27.1 | 0.3 | 22.2 | 15.9 | 16.4 | 56.9 | 46.2 | 50.2 | 123.9 | 110.8 | 117.6 | 179.0 | 165.1 | 173.0 | 18.1 | 17.2 | 18.0 | 61.8 | 61.1 | 61.2 | 7.57 | 5.36 | 5.94 |
| **9** | 40 | 37.2 | 6.0 | 12.8 | 6.4 | 7.2 | 47.6 | 38.2 | 38.2 | 83.8 | 70.8 | 70.2 | 101.9 | 105.3 | 102.2 | 14.0 | 15.1 | 15.4 | 61.8 | 61.0 | 67.4 | 2.73 | 1.86 | 1.72 |
| **10** | 251.3 | 218.6 | 80.1 | 4.1 | 0.79 | 1.9 | 15.5 | 12.1 | 12.9 | 32.8 | 30.4 | 30.9 | 42.8 | 43.3 | 43.9 | 16.3 | 13.4 | 13.4 | 62.6 | 60.9 | 64.6 | 1.51 | 0.83 | 1.01 |
| **11**‡ | 107.9 | 100.7 | 6.24 | 0 | 0 | 0 | 0 | 0 | 0 | 0 | 0 | 0 | 0 | 0 | 0 | 13.0 | 11.4 | 11.7 | 23.4 | 23.6 | 24.8 | 0.00 | 0.00 | 0.00 |
| **12** | 70.5 | 71.6 | 11.4 | 35.9 | 17.8 | 19.4 | 71.4 | 60.6 | 62.5 | 119.5 | 103.5 | 106.1 | 141.0 | 142.4 | 145.0 | 17.5 | 18.4 | 18.7 | 62.7 | 61.4 | 65.4 | 6.16 | 3.83 | 4.20 |

§GTV, the gross extent of the tumor before radiotherapy; *GTV2, residual abnormal shadows in CT images at 40 Gy; †BTV**,** the regions with SUV above 2.0

‡patient with lateral pelvic lymph node metastasis or perineum recurrence

**Table 2: Iirradiated volume or dose of PRV and NTCP in each summed** plan

|  | ***summed plan1***  **(CRT)** | ***summed plan2***  **(IMRT without dose-paint)** | ***summed plan3***  **(IMRT with dose-paint)** |
| --- | --- | --- | --- |
| **V_30Gy_ (cm^3^ )** | 121.18±119.68 | 113.62±99.69 | 116.88±104.94 |
| **V_40Gy_ (cm^3^ )** | 77.32±64.21 | 71.33±60.20 | 72.55±61.59 |
| **V_50Gy_ (cm^3^ )*** | 47.11±45.33 | 40.63±39.13 | 41.25±39.96 |
| **V_60Gy_ (cm^3^ )**† | 19.76±23.67 | 13.65±18.88 | 14.52±19.18 |
| **D_mean_ (Gy)**† | 16.1±5.8 | 16.4±5.6 | 16.6±5.8 |
| **D_max_ (Gy)††** | 55.0±16.0 | 54.9±15.3 | 57.4±16.3 |
| **NTCP (%)**† | 5.10±5.66 | 3.78±4.19 | 4.09±4.62 |

***** V_50Gy_ of PRV in *summed plan1* was significantly larger than that in *summed plan2* and *summed plan3*.

†With regard to V_60Gy_, D_mean_ and NTCP of PRV, there were no significant differences.

**††**D_max_ of PRV in *summed plan3* is significantly higher than that in *summed plan 1* and *summed plan2.*
